# Circulating IL-27 Is Elevated in Rheumatoid Arthritis Patients

**DOI:** 10.3390/molecules21111565

**Published:** 2016-11-18

**Authors:** Xiaofei Lai, Hongxu Wang, Ju Cao, Ying Li, Yubing Dai, Yu Xiang, Liping Zhang

**Affiliations:** 1 Department of Laboratory Medicine, The First Affiliated Hospital of Chongqing Medical University, Chongqing 400016, China; lxf224234@163.com (X.L.); wanghx502@163.com (H.W.); caoju723@163.com (J.C.); suipian8311@sina.com(Y.L.); 2 Center for Nuclear Receptors and Cell Signaling, University of Houston, Houston, TX 77204, USA; ybdai@central.uh.edu

**Keywords:** IL-27, rheumatoid arthritis, inflammation, immune response, disease activity

## Abstract

Cytokines are key immunoregulatory molecules that regulate T lymphocyte-mediated immune responses and inflammatory reactions. We determined whether there is aberrant expression of interleukin-27 (IL-27) in rheumatoid arthritis (RA) patients and investigated the clinical significance of these changes. IL-27 is a key cellular factor that regulates the differentiation of CD4+ T cells, which can secrete interleukin-10 (IL-10) and interleukin-17 (IL-17) in vivo. Concentrations of serum IL-27 in 67 RA patients, and 36 sex- and age-matched control subjects were measured by enzyme-linked immunosorbent assay (ELISA). Results showed that concentrations of serum IL-27 in all RA patients were significantly higher than in healthy control subjects, and there was a significant and positive correlation between serum IL-27 levels and disease activity in all RA patients. Levels of serum IL-27 in RA patients were significantly correlated with disease activity score in 28 joints (DAS28). Moreover, immunosuppressive treatment with leflunomide downregulated the levels of IL-27 in active RA patients. Therefore, the elevated production of circulating T cell inflammatory factors contributes to the pathogenesis of RA, and serum IL-27 could potentially serve as a new biomarker of RA disease activity.

## 1. Introduction

Rheumatoid arthritis (RA) is an autoimmune disease that is mainly characterized by chronic inflammation and destruction of the joints [1]. RA is associated with various immunological abnormalities, such as increased numbers of activated T lymphocytes and aberrant expression of inflammatory cytokines [2,3]. The various pro-inflammatory cytokines secreted by infiltrating macrophages, T and B cells in the synovial fluids and tissues contribute to joint inflammation [4] and play crucial roles in both joint damage and propagating inflammation in RA [5]. Autoimmune diseases are associated with dysregulated immune responses, and it has been suggested that increased expression of immune modulators could facilitate the activation and survival of the inflammatory cells that mediate the development of autoimmune diseases [6,7].

The etiology and pathogenesis of RA is not yet fully understood, but it has been confirmed that dysregulated expression of inflammatory cytokines contributes to its occurrence, persistent inflammation and joint destruction. Interleukin (IL)-27 is a novel member of the IL-6/IL-12 family of cytokines that is produced by antigen-presenting cells [8]. Recent studies have described IL-27 as a promoter of Th1 differentiation in naive CD4+ T cells. IL-27 also has broad inhibitory effects on Th1, Th2 and Th17 T cells. However, it expanses the population of inducible regulatory T cells (Tregs) [9,10]. IL-27 is a heterodimeric cytokine consisting of EBI3, an IL-12 p40 homologue originally described to be secreted by Epstein–Barr-virus-transformed B cells, and p28, an IL-6 and p35 homologue [11,12]. There are a range of cells that express the IL-27 receptor, which is a heterodimer, composed of IL-27 Ralpha and the protein gp130. The IL-27R is expressed on naive T cells, mast cells, endothelial cells etcetera [13,14,15]. In innate immunity, IL-27 can provoke the production of IL-1β, TNF-α, IL-18 and IL-12 in monocytes [16]. However, more recent studies revealed that IL-27 can also play a regulatory role of suppressing the acquired immunity and expanding the population of inducible Tregs that produce IL-10 [9,10]. Additionally, in collagen-induced arthritis (CIA) mouse models, administration of IL-27 attenuated the severity of the disease and suppressed monocyte recruitment and angiogenesis [17]. Although previous reports have shown that IL-27 plays a critical role in the pathogenesis of RA, the molecular basis of the pleiotropic actions of IL-27 in various immune responses has not been well elucidated. In this study, we investigated the presence and significance of circulating IL-27 in RA patients to evaluate its potential as a disease biomarker for RA.

## 2. Results

### 2.1. RA Patients and Control Subjects

Clinical and demographic information, including age, sex, duration of diagnosis, rheumatoid factors (RF), anti-cyclic cirullinated peptide antibodies (anti-CCP), erythrocyte sedimentation rate (ESR), C-reactive protein (CRP), white blood cell (WBC), hemoglobin (Hb), platelet(PLT), Leukocyte %, Neutrophile % and DAS28, T28, SW28 of the study populations are summarized in Table 1. There were 67 RA patients and 36 sex- and age-matched control subjects. The mean time of RA disease duration (since the diagnosis of RA to the time when patients were recruited for this study) was 9.6 ± 3.5 years. The DAS28 scores of RA patients were 5.5 ± 1.1. The serum RF and anti-CCP levels were significantly elevated in the RA group compared with the control group (*p* < 0.001). Meanwhile, CRP and ESR and leukocytes were also significantly higher in RA patients than healthy controls (*p* < 0.001). However, hemoglobin (Hb) concentration and platelets (PLT) were decreased.

### 2.2. Serum Levels of IL-27

**Figure 1:**
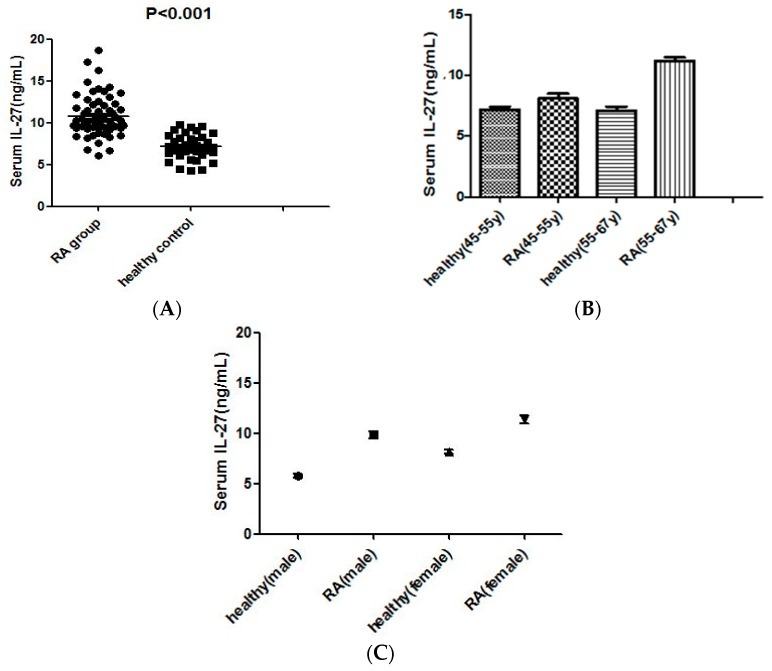
(**A**) scatter-plots of concentrations of serum interleukin-27 (IL-27) in healthy control subjects and rheumatoid factors (RA) patients. The horizontal lines indicate the median concentration for each molecule. The differences between RA patients and controls were determined by non-parametric Mann–Whitney rank sum test; (**B**) scatter-plots of concentrations of serum IL-27 in different age groups. (*p* > 0.05 healthy (45–55) vs. healthy (56–67)); and (**C**) scatter-plots of concentrations of serum IL-27 in different sex groups. (*p* < 0.001 female vs male).

Serum levels of IL-27 were measured in RA patients and healthy controls by ELISA. As shown in Figure 1A, IL-27 was ubiquitously present in human serum, and IL-27 concentrations were significantly higher in RA patients than in control subjects (10.7 (6.2–11.1) vs. 6.2 (4.2–8.9)) (*p* < 0.001). The levels of serum IL-27 increased with age in RA patients. However, this phenomenon did not exist in the healthy group (Figure 1B). The difference was performed in female and male (Figure 1C).

### 2.3. Serum Concentration of IL-27 and RA Disease Activity

**Figure 2:**
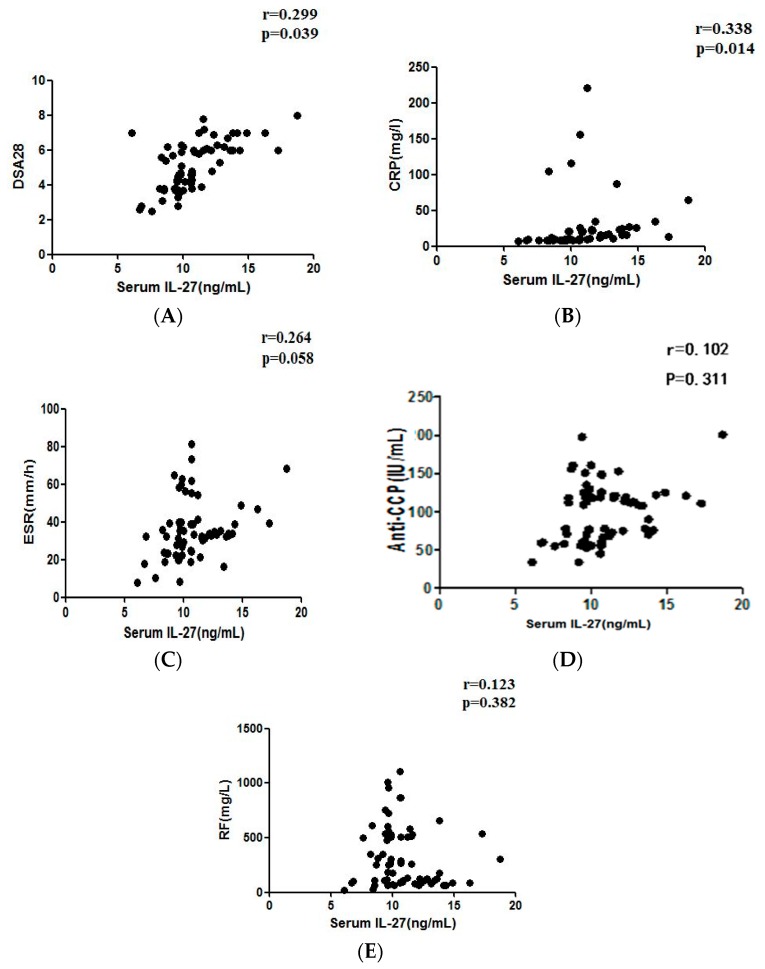
(**A**) Correlation of serum IL-27 concentrations with disease activity score in 28 joints (DAS28) in all RA patients (*n* = 67). Spearman's rank correlation coefficient was employed to assess correlations; (**B**) correlation of serum IL-27 concentrations with C-reactive protein (CRP) in all RA patients (*n* = 67). Spearman’s rank correlation coefficient was employed to assess correlations; *p*-values are shown; (**C**) correlation of serum IL-27 concentrations with erythrocyte sedimentation rate (ESR) in all RA patients (*n* = 67). Spearman’s rank correlation coefficient was employed to assess correlations; *p*-values are shown; (**D**) correlation of serum IL-27 concentrations with anti-cyclic cirullinated peptide antibodies (anti-CCP) in all RA patients (*n* = 67). Spearman’s rank correlation coefficient was employed to assess correlations; *p*-values are shown; and (**E**) correlation of serum IL-27 concentrations with rheumatoid factors (RF) in all RA patients (*n* = 67). Spearman’s rank correlation coefficient was employed to assess correlations; *p*-values are shown.

Concentrations of serum IL-27 exhibited a positive and significant correlation with DAS28 scores in RA patients (r = 0.299, *p* = 0.039; Figure 2A). The positive correlation persisted even when we redefined RA patients using DAS28 scores of 4 and 5 (instead of 3) as the cut-off values (all *p* < 0.05; data not shown). DAS28, T28 and SW28 were higher in active RA patients than inactive subjects. (*p* < 0.05; in Table 2).

### 2.4. Correlation between Serum IL-27 and RF, Anti-CCP, CRP or ESR Levels in RA Patients

As shown in Figure 2B, levels of serum IL-27 significantly correlated with serum CRP concentrations (r = 0.338, *p* = 0.014). However, there was no significant correlation between serum IL-27 and ESR, anti-CCP, or RF levels in RA patients (all *p* > 0.05, Figure 2C–E).

### 2.5. Effect of Immunosuppressant Treatment on the Production of IL-27

**Figure 3:**
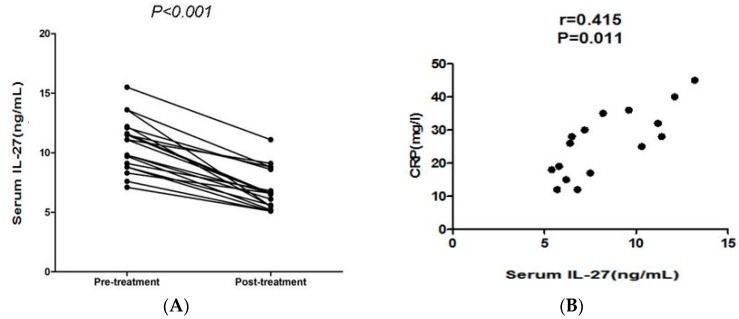

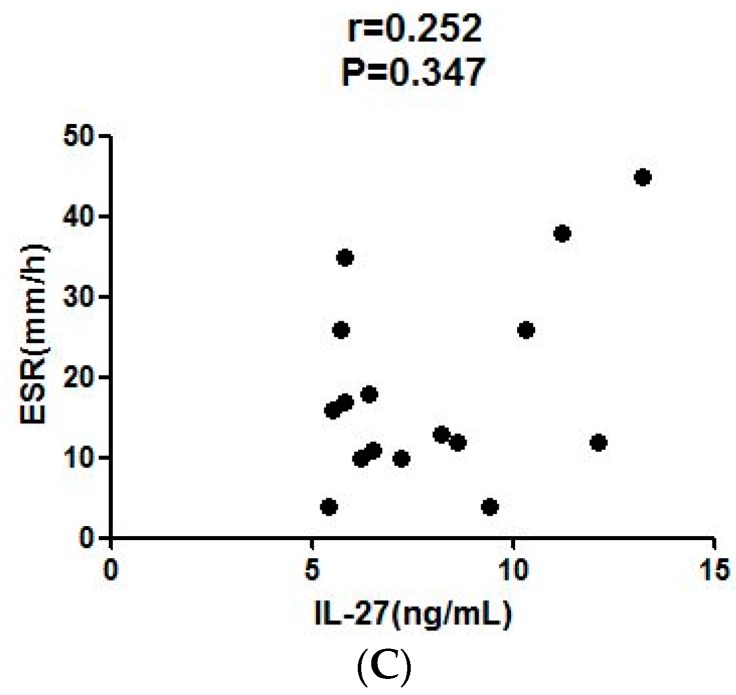
(**A**) Serum IL-27 levels were significantly decreased after RA amelioration with leflunomide; (**B**) the correlation of IL-27 concentrations with CRP in the RA patients after leflunomide (LEF) treatment; and (**C**) the correlation of IL-27 levels of ESR in the RA patients after LEF treatment.

Sixteen RA patients with disease activity were recruited after an eight-week treatment with leflunomide (LEF), and we found that serum IL-27 level in all 16 patients were significantly decreased by eight-week treatment (*p* < 0.01, Figure 3A). IL-27 levels were decreased by > 30% in most RA patients. Moreover, levels of CRP, anti-CCP and RF, ESR, leukocytes, and scores of HAQ, T28, SW28 and DAS28 in 16 active RA patients were significantly decreased after eight-week LEF treatment (*p* < 0.05, Table 2).

### 2.6. Correlation of Serum IL-27 to Parameters after LEF Treatment 

As illustrated in Table 2, levels of serum IL-27 significantly correlated with leukocytes, Hb, levels of serum CRP and RF, and scores of HAQ, T28, SW28, DAS28 (all *p* < 0.05). However, there was no significant correlation between serum IL-27 and ESR, and anti-cyclic cirullinated peptide antibodies (anti-CCP) in RA patients (all *p* > 0.05).

## 3. Discussion

In recent years, many studies have revealed that cytokines play pivotal roles in the pathogenesis of RA. IL-27 is mainly produced by antigen-presenting cells and regulates T differential and function of T cells [18]. Moreover, IL-27 could modulate expression of IL-10 and IL-17 in CD4+ T cells and promote the differentiation of Treg cells and inhibit the generation of TH17 cells [19]. Thus, in autoimmune and inflammatory reactions, IL-27 could act as an inflammatory or anti-inflammatory effector [20]. Recent studies have found that IL-27 could only inhibit the early differentiation stages of Th17 cells, instead of differentiation of mature Th17 cells. Thereby, the efficacy of treatment with IL-27 to autoimmune diseases in which IL-27 Th17 cells participate may be insignificant. Nevertheless, IL-27 can suppress Th2-type immune responses [21]. Previous studies demonstrated that IL-27 induced Th1 response, and activated T cells, nature killer (NK) cells, mast cells and mononuclear cells to produce IFN-γ, IL-1, IL-12, IL-18 and TNF-α inflammatory cytokines in early stages of inflammatory responses [22].

Platelet count was significantly decreased in the active RA patients group compared with healthy controls. Platelets were identified as vital regulators in autoimmune diseases, especially in RA [23,24,25]. Platelets could amplify inflammation in arthritis via collagen-dependent microparticle production [24,26]. Hyperactive platelets are also responsible for arthritic and cardiovascular implications in RA patients [27]. Platelet activation contributes, along with classical sources, to elevated plasma IL-27 levels [28]. Moreover, mean platelet volume (MPV) reflects disease activity of RA [29,30]. In addition, the role of Myeloid derived suppressor cells (MDSCs) in Tregs and Th17 cell expansion was inconsistent. Given their role in suppressing T-cell responses, MDSCs have been tested for their capability of preventing RA [31]. Meanwhile, IL-27 could regulate Treg and Th17 cell expansion. Thus, we supposed that MDSCs may have a relationship with IL-27, though this needs further verification.

In this study, we demonstrated that serum concentrations of IL-27 in RA patients were significantly higher than those of control subjects, which is consistent with previous studies [32,33,34]. Apart from clinical parameters of DAS28, RF, CCP, ESR, CRP enrolled in the previous study [33], we added other features such as WBC, PLT, Hb, etc. Additionally, we focused on the alteration of serum IL-27 pre- and post-treatment. Serum IL-27 levels of 16 active RA patients were significantly decreased in after eight weeks of LEF treatment. Leflunomide was approved by the Food and Drug Administration for the treatment of rheumatoid arthritis (RA) based on double-blind, multicenter trials [35]. LEF is an immunomodulatory drug that may exert its effects by inhibiting the mitochondrial enzyme dihydroorotate dehydrogenase (DHODH), which plays a key role in the de novo synthesis of the pyrimidine ribonucleotide uridine monophosphate (rUMP). Briefly, LEF prevents the expansion of activated and autoimmune lymphocytes (mainly activated T lymphocytes) by interfering with the cell cycle progression [36]. LEF caused a decrease of levels of TNF, MMP-1, MMP-3, IL-6 and IL-10 [37,38]. Therefore, LEF treatment may regulate serum levels of inflammatory cytokine. Since TNF is a major inducer of IL-27 in antigen presenting cells, we hypothesize that anti-TNF treatment will also downregulate IL-27 in RA patients, but further studies are needed. IL-27 regulates activity of B- and T-lymphocytes. The presence of elevated serum IL-27 levels suggest that IL-27 may play important roles in immunological derangements of T and B cells in RA. However, it is still unclear whether elevation of serum IL-27 level in RA contributes to RA pathogenesis.

Apart from plasma IL-27, RA patients also had significantly elevated levels of anti-CCP, ESR, and CRP (Table 1). However, they were not ideal biomarkers for disease activity of RA. The positive rate of anti-CCP in RA patients is nearly 80%. In many inflammatory conditions, infectious or autoimmune reactive, levels of ESR and CRP non-specifically elevated.

Previous studies have uncovered that treatment with exogenous IL-27 could alleviate arthritic inflammation and inhibit the defined mediators of inflammation, angiogenesis, cell survival, apoptosis, and tissue damage [39,40]. Additionally, IL-27 may negatively regulate development of ectopic lymphoid-like structures in RA through controlling effector T cells [40]. Our study unveiled that there is a coherent correlation of IL-27 concentrations with a DAS28 score. Our research data indicated that levels of circulating serum IL-27 could potentially serve as a surrogate biomarker of RA disease activity. To investigate clinical functions of IL-27 more specifically, studies with larger cohorts of RA patients are demanded.

## 4. Materials and Methods

### 4.1. Subjects

A total of 67 patients who fulfilled the American College of Rheumatology criteria for RA were included in the study. The group of RA patients included 39 females and 28 males. There were 23 active RA patients in those 67 patients. The RA patients were not complicated with other autoimmune, inflammatory and tumor diseases. The demographic and key clinical information of the patients is summarized in Table 1. Patients included in this study were recruited from the First Affiliated Hospital of Chongqing Medical University and did not receive any immuno-suppressive or anti-inflammatory drugs for any condition within at least two months before sample collection. The health assessment questionnaire and disease activity score in 28 joints (DAS28) were completed for all patients. Thirty-six age-matched healthy Chinese volunteers were recruited as normal control subjects. Written informed consent was obtained from each individual, and the protocol was approved by the Clinical Research Ethics Committee of the First Affiliated Hospital of Chongqing Medical University, and informed consent was obtained from all participants according to the Declaration of Helsinki. Among 23 active RA patients, 16 patients received mono-therapy of leflunomide (20 mg/day) for eight weeks. The rest of the seven active RA patients receiving other therapy regimens were excluded from the observation group.

### 4.2. Measurement of Auto-Antibodies 

Rheumatoid factor (RF)-IgM was measured using a Beckman Coulter Immunoge800 auto-analyzer (Beckman Coulter Inc., Jersey City, NJ, USA) in accordance with the supplied instructions. Serum concentrations of C-reactive protein (CRP) were also determined by auto-analyzer (Beckman Coulter Inc.). The whole blood erythrocyte sedimentation (ESR) was manually performed via the Westergren method. WBC, Hb, PLT, Leukocyte %, and Neutrophile % were also determined by an auto-blood cell analyzer (Sysmex, Kobe Tokyo, Japan).

Serum levels of anti-cyclic citrullinated peptide (anti-CCP) were measured using an ELIA™ CCP test system (Phadia GmbH, Freiburg, Germany), and samples were analyzed with an ImmunoCAP100 instrument (Phadia GmbH, Freiburg, Germany).

### 4.3. Assay of IL-27 

Concentrations of serum IL-27 were measured by ELISA (Bender Medsystems Diagnostics, Vienna, Austria).

### 4.4. Statistical Analysis 

IL-27 concentrations did not have a Gaussian distribution; therefore, the Mann–Whitney rank sum test was used to analyze the differences in IL-27 concentrations between patients and controls, and the Spearman's rank correlation test was used to assess the correlations of serum IL-27 concentrations with DSA28, RF, anti-CCP, CRP and erythrocyte sedimentation rate (ESR) levels. Results are expressed as median (interquartile range). All analyses were performed using the Statistical Package for the Social Sciences (SPSS) statistical software, version 9.0 (SPSS, Chicago, IL, USA). *p* < 0.05 was considered a significant difference.

## 5. Conclusions

In conclusion, our study demonstrated that levels of circulating serum IL-27 were elevated in RA patients and were positively correlated with RA disease activity. Moreover, concentrations of serum IL-27 decreased after effective immunosuppressant treatment. Probably, IL-27 is involved in the pathogenesis of RA and could be a potential biomarker for RA disease activity. These findings shed new light on the dysregulation of the immune system in autoimmune diseases.

## Figures and Tables

**Table 1:** The correlation of interleukin-27 (IL-27) and clinical characteristics after eight-week treatment [95% confidence interval (CI)].

Features	Pre-Treatment	Post-Treatment	*p* *	Correlation	
				r	*p*
T28 (IQ range)	15.5 (12.4–17.8)	6 (3.7–8.7)	0.000	0.886	0.000
SW28	8.5 (7.1–10.7)	1.5 (1.1–2.8)	0.092	0.614	0.011
WBC (×10^9^/L)	5.8 (4.5–6.5)	6.2 (5.4–6.9)	0.000	0.610	0.012
Leukocyte %	55 (48.9–57.6)	49 (43.4–50.4)	0.001	0.845	0.000
Neutrophile %	40 (38.5–43.1)	45 (44–46)	0.007	0.542	0.03
PLT (×10^9^/L)	263 (256–293)	271 (248–288)	0.176	0.227	0.397
Hb (g/L)	119 (113.8–125.2)	117 (111.2–122.4)	0.000	0.828	0.000
RF, IU/mL	134.2 (61.3–331.8)	49 (37–77)	0.021	0.704	0.002
Anti-CCP, U/mL	85.5 (88.7–102.9)	11.5 (9.7–15.7)	0.157	0.126	0.643
ESR, mm/h	31.5 (27.5–37.3)	14.5 (12.0–25.0)	0.015	0.252	0.347
CRP, mg/L	22 (16.8–45.3)	13.5 (12.2–22.4)	0.004	0.415	0.11
HAQ	7 (6.2–7.2)	2.8 ± 1.1	0.000	0.893	0.000
DAS28	5.8 ± 1.2	4 (3.3–4.9)	0.000	0.934	0.000

T28, Tender joint count in 28 joints; SW28, swollen joint count in 28 joints; WBC white blood cells; PLT, platelets; Hb, hemoglobin; RF, rheumatoid factors; Anti-CCP, anti-cyclic cirullinated peptide antibodies; ESR, erythrocyte sedimentation rate; CRP, C-reactive protein; HAQ, health assessment questionnaire; DAS28, disease activity score in 28 joints. *p* * < 0.001 vs control; correlation analyses of serum IL-27 levels and the parameters in disease activity after eight weeks of leflunomide.

**Table 2:** Demographic and clinical characteristics of the study subjects (95% CI).

Features	Healthy Controls (*n* = 36)	Rheumatoid Arthritis Group (*n* = 67)	Active Patients (*n* = 23)
Age, years	57.6 (45.5–66.1)	55.2 (48.1–66.7)	41 (40.3–44.4)
Male/female	15/21	28/39	6/17
Disease duration, years	No	9.6 ± 3.5	8 ± 1.5
RF, IU/mL	12 (1–21)	311.0 (83.1–1111.0) *	88 (47.9–24.2)
Anti-CCP, U/mL	7 (0–19)	111.6 (68.5–186.5) *	79 (89.5–96.2)
ESR, mm/h	7 (5–15)	28.1 (15.0–34.2) *	31 (28.3–35.0)
CRP, mg/L	1.11 (0.4–2.1)	14.6 (3.6–48.5) *	17 (15.8–36.1)
DAS28	No	5.5 ± 1.1 *	5.8 ± 1.3 *
IL-27 (ng/mL)	6.2 (4.2–8.9)	10.7 (6.2–11.1) *	9.4 (8.5–12.2)
T28	0	8 (6.6–13.2)	14 (12.3–16.5) ^●^
Sw28	0	5.2 (4.2–9.8)	9.7 (8.3–10.9) ^●^
WBC (×10^9^)	5.8 (4.8–7.2)	5.5 (4.9–6.3) *	5.2 (4.7–5.9)
Leukocyte %	46 (41.9–53.9)	47.3 (40.1–48.9) *	48 (45.9–52.3)
Neutrophile %	58 (50.9–62.3)	48 (46.9–60.1) *	45 (43.6–50.6)
PLT (×10^9^)	302 (290.3–311.3)	285 (265.3–288.2) *	278 (270.1–292.5)
Hb (g/L)	127.3 (120.4–131.5)	118 (117.6–123.3) *	115 (111.9–119.7)
HAQ	0	6 (2.7–6.5)	7 (4.7–5.6)

RF, rheumatoid factors; Anti-CCP, anti-cyclic cirullinated peptide antibodies; ESR, erythrocyte sedimentation rate; CRP, C-reactive protein; DAS28, disease activity score in 28 joints; IL-27 interleukin-27; T28, Tender joint count in 28 joints; Sw28, swollen joint count in 28 joints; WBC white blood cells; PLT, platelets; Hb, hemoglobin; HAQ, health assessment questionnaire. * RA group *p* < 0.001 vs. control, ^●^ active group *p* < 0.001 vs. RA group.
